# Influence of Low-Frequency Vibration and Modification on Solidification and Mechanical Properties of Al-Si Casting Alloy

**DOI:** 10.3390/ma10050560

**Published:** 2017-05-20

**Authors:** Vadim Selivorstov, Yuri Dotsenko, Konstantin Borodianskiy

**Affiliations:** 1 Electrometallurgical Faculty, The National Metallurgical Academy of Ukraine, 4 Gagarina Ave., Dnipro 49600, Ukraine; s-v-y@mail.ru (V.S.); yuri.dotsenko1@gmail.com (Y.D.); 2 Zimin Advanced Materials Laboratory, Department of Chemical Engineering, Biotechnology and Materials, Ariel University, Ariel 40700, Israel

**Keywords:** aluminum casting alloy, permanent mold, vibration treatment, modification, mechanical properties

## Abstract

One of the major aims of the modern materials foundry industry is the achievement of advanced mechanical properties of metals, especially of light non-ferrous alloys such as aluminum. Usually an alloying process is applied to obtain the required properties of aluminum alloys. However, the presented work describes an alternative approach through the application of vibration treatment, modification by ultrafine powder and a combination of these two methods. Microstructural studies followed by image analysis revealed the refinement of α-Al grains with an increase in the Si network area around them. As evidence, the improvement of the mechanical properties of Al casting alloy was detected. It was found that the alloys subjected to the vibration treatment displayed an increase in tensile and yield strengths by 20% and 10%, respectively.

## 1. Introduction

In recent years, the materials manufacturing industry has become more and more interested in aluminum alloys production, especially Al-Si alloys. This is due to the advanced properties of such alloys, such as high thermal and electrical conductivity, and relatively low density which is, only one third of that of steel [1]. Unfortunately, aluminum alloys still show relatively lower mechanical properties compared to those of iron-based alloys. Traditionally, strengthening of Al is achieved by an alloying process, involving the addition of different compounds that affect the metal structure formation and, as a result, influence its mechanical properties.

Al-Si hypoeutectic casting alloys are commonly used in automotive and aerospace industries due to their high strength at elevated temperatures. Therefore, investigation of these alloys’ mechanical properties is of great interest in the light alloy foundry industry. Mostly, the alloys’ strength improves by the alloying approach [2,3,4,5,6,7]. Such works also demonstrate the mechanical properties improvement using the ultrasound method, which affects the metal solidification process [8,9,10].

Over the last two decades, we have found that the improvement of metals’ mechanical properties can be achieved by a method that involves the addition of various nanomaterials into the melt. Several published works pointed out that the mechanical properties of A356 alloy can be improved by the addition of Al_2_O_3_, TiB_2_, TiC or WC nanoparticles during the casting process [11,12,13,14].

Vibration treatment is an additional approach which is widely applied during metal solidification to improve their macro- and microstructures and consequently their mechanical properties. The majority of these works explain the effect of vibration treatment and attribute it to the cavitation phenomenon [15,16]. Jian et al. described a significant refinement of the microstructure of aluminum A356 alloy by ultrasonic vibration [17]. Later, Gencalp and Saklakoglu illustrated the microstructural changes of Al A380 alloy as the result of the vibration frequency changing [18]. Identical microstructural behavior has been also described by Limmaneevichitr and co-workers. Here, more globular and finer microstructures of Al A356 alloy were obtained as the vibration increased [19]. The similar effect of obtaining small globular aluminum grains was also described in the investment casting of Al alloy by Barbosa and Puga [20].

In some works, we can even find the improvement of physical and mechanical properties simultaneously, e.g., Okayasu et al. showed the effect of ultrasonic vibration on Al casting alloys and pointed out that the electric conductivity was about 10% higher compared to wrought Al alloy, while at the same time the treatment improved the mechanical properties of the Al casting alloy [21].

The object of the current work is study of the vibration treatment, modification by ultrafine particles, and a combined treatment approach on the solidification effect and mechanical properties of Al-Si casting alloy. Through image analysis, macro- and microstructures will be investigated, as well as the mechanical properties of the obtained alloys. Additionally, X-ray diffraction (XRD) studies will be performed to reveal any changes in the phase composition of the alloy.

## 2. Materials and Methods

Commercial aluminum 356 casting alloy was used as the bulk material. The composition of the alloy is given in Table 1.

**Figure 1:**
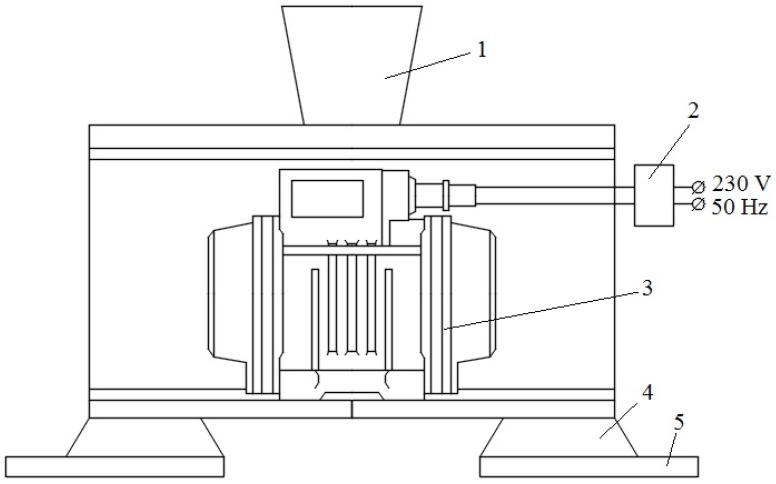
Schematic illustration of the vibration treatment device: 1. Casting mold; 2. Frequency converter; 3. Vibrator exciter; 4. Support; 5. Fixation system.

A 100 kg portion of ingots of 356 alloy was melted in an industrial electric resistance furnace and superheated up to 680 °C, and then subjected to the addition of commercial degassing compound Degasal T200 (Schaefer Chemiche Fabrik, Hennef (Sieg), Germany) to clean the melt from the oxides and hydrogen. After 10 min, the melt was poured into a cast iron permanent mold with an average diameter of 60 mm, a wall thickness of 5 mm, and a height of 150 mm. First, the mold was preheated up to 400 °C and then the melt was poured at 720 °C ± 5 °C. The pouring process was applied with various vibration frequencies, 100 Hz, 150 Hz, and 200 Hz, and with the same amplitude of 0.7 mm; the schematic illustration of the device shown in Figure 1. Additionally, the as-cast, untreated alloy was poured for comparison.

A combined treatment of the modification approach followed by the vibration treatment was made by a specially prepared homemade “Typhoon-Z” modifier, which is based on the mixture of ultrafine oxide powders in the size range of 3–5 μm; its composition is presented in Table 2.

0.1 wt % of the modifier was added into the melt and, after 10 min of holding, the melt was poured and subjected to the vibration treatment. For comparison reasons, a modified alloy which was not subjected to the vibration treatment was poured into the mold as well. Table 3 presents the samples under different conditions performed in the experimental research.

Microscopic examinations were carried out using an Olympus BX53MRF-S (Tokyo, Japan) optical microscope. All specimens were analyzed after etching by Keller-Wilcox’s reagent (3 mL HCl, 5 mL HNO_3_, 1 mL HF, and 190 mL H_2_O). For measuring the size of the α-Al grains and a percentage of the eutectic phase area, Clemex image analysis software (Longueuil, QC, Canada) was applied.

**Figure 2:**
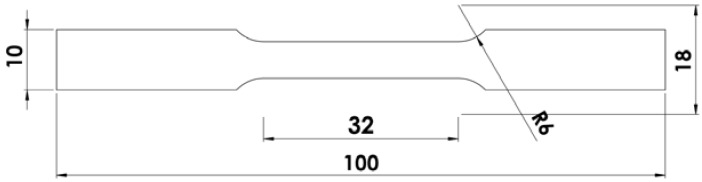
Schematic illustration of the specimen subjected to mechanical properties tests according to ASTM B 108-1.

The mechanical properties were measured by a testing machine FP 100 (Heckert, Germany) according to the ASTM B 108-01. Comparisons were made between the as-cast alloy, the modified alloy by a homemade modifier, the alloy after vibration treatment, and the alloy after a combined treatment using vibration and modification. A schematic illustration of the specimen subjected to mechanical properties tests is shown in Figure 2.

Macrostructure and shrinkage defects were analyzed by the obtained solidified ingots. Each ingot was cut vertically in order to present the formation of the complete cast.

Phase analysis of the modified and unmodified alloys was identified by a Panalytical X’Pert Pro X-ray powder diffractometer (Panalytical, Almelo, The Netherlands) at 40 kV and 40 mA. The patterns were measured and recorded at the 2Θ range from 20° to 100° (step size/time per step: 0.02°/1 s).

The density of the aluminum alloys was determined by a hydrostatic weighing approach with the accuracy of 0.001 g/cm^3^. Crack- and cavity-free samples were weighed in air and in carbon tetrachloride (CCl_4_) by using analytical scales. The density of the alloys was calculated by the following equation:

(1) $$d_{s}=\frac{P_{a}}{P_{a}-P_{h}}\cdot \left(d_{h}-0.0012\right)+0.0012,$$

where

*d_s_*—density of the sample (g/cm^3^);
*Р_a_*—mass of the sample in the air (g);
*Р_h_*—mass of the sample in ССl_4_ (g);
*d_h_*—density of the CCl_4_ (g/cm^3^).

## 3. Results and Discussion

**Figure 3:**
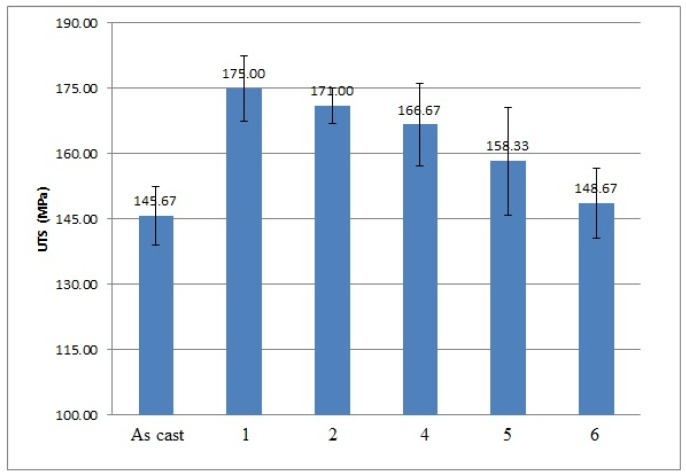
Results of ultimate tensile strength (UTS) measurements performed on 356 Al alloy: 1—after vibration by 100 Hz; 2—after vibration by 150 Hz; 4—after modification by ultrafine powder modifier; 5—after modification by ultrafine powder modifier followed by 100 Hz vibration; 6—after modification by ultrafine powder modifier followed by 150 Hz vibration; As cast—unmodified and untreated alloy.

**Figure 4:**
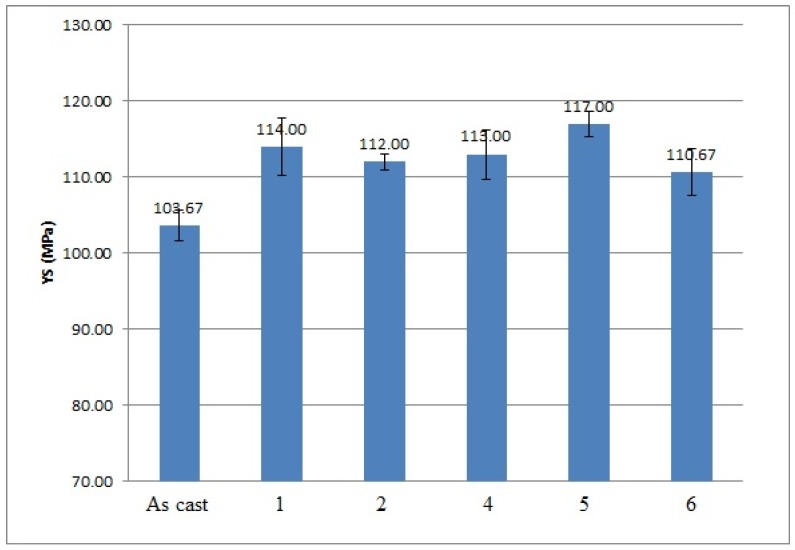
Results of yield strength (YS) measurements performed on 356 Al alloy: 1—after vibration by 100 Hz; 2—after vibration by 150 Hz; 4—after modification by ultrafine powder modifier; 5—after modification by ultrafine powder modifier followed by 100 Hz vibration; 6—after modification by ultrafine powder modifier followed by 150 Hz vibration; As cast—unmodified and untreated alloy.

**Figure 5:**
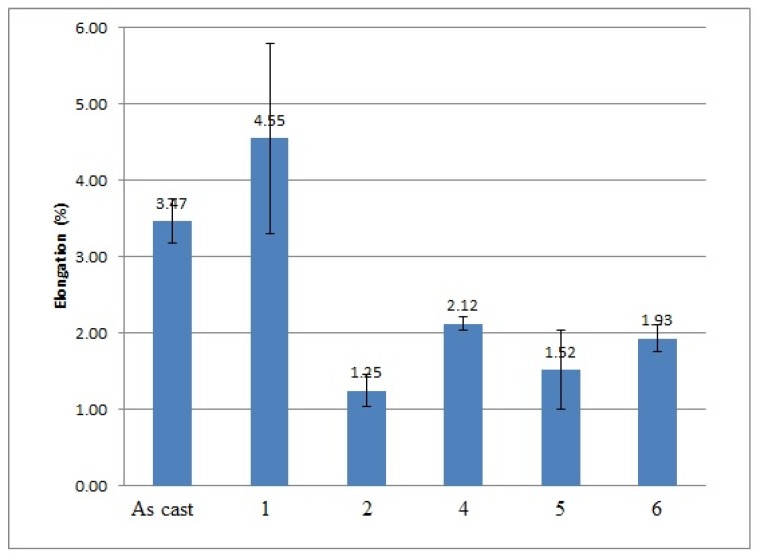
Results of elongation measurements performed on 356 Al alloy: 1—after vibration by 100 Hz; 2—after vibration by 150 Hz; 4—after modification by ultrafine powder modifier; 5—after modification by ultrafine powder modifier followed by 100 Hz vibration; 6—after modification by ultrafine powder modifier followed by 150 Hz vibration; As cast—unmodified and untreated alloy.

The effects of applying low frequency vibration alone and vibration jointly with the modification process on the mechanical properties of Al 356 alloy are shown in Figure 2, Figure 3, Figure 4 and Figure 5.

The mechanical properties of the alloy that was subjected to 200 Hz vibration treatment were not fully determined because this approach resulted in low-quality cast samples due to major defects such as cavities and high porosity.

As it is shown in the presented mechanical properties measurements, the tensile and yield strengths of all alloys after the vibration treatment and modification were slightly improved. The greatest ultimate tensile strength (UTS) results were obtained after 100 Hz and 150 Hz vibration, they improved by 20% and 17% respectively; their yield strength was improved as well by 10% and 8%, respectively. The yield strength of the alloy subjected to the combined treatment by 100 Hz and modification was even improved by 13%. The elongation of all alloys mainly decreased, which shows the usual metals’ behavior when an increase in the strength reduces the ductility of the metal and vice versa. The elongation values of the alloy subjected to the 100 Hz vibration were unexpected; the obtained results are unstable and have a large range in values due to macro-segregation effects in this cast, which occur between the surface and the center of the cast. Moreover, large pores were indicated in the fracture surface after the mechanical tests, which is a possible reason for the extremely high elongation values in this sample. The density measurements are presented in Table 4.

Based on results in Table 4, it is evident that the density values of the alloys vary considerably, ranging between 2.729 g/cm^3^ and 2.852 g/cm^3^, especially in samples subjected to the vibration treatment. For example, the density of the alloy subjected to the vibration treatment by 150 Hz, untreated alloy, and modified alloy only, is within the ranges of 2.750 g/cm^3^–2.852 g/cm^3^ (3.7% difference), 2.739 g/cm^3^–2.773 g/cm^3^ (1.2% difference), and 2.736 g/cm^3^–2.737 g/cm^3^ (0.04% difference), respectively. The main reason for the high differences in the densities subjected to the vibration treatment is the presence of segregation areas in the obtained ingots.

**Figure 6:**
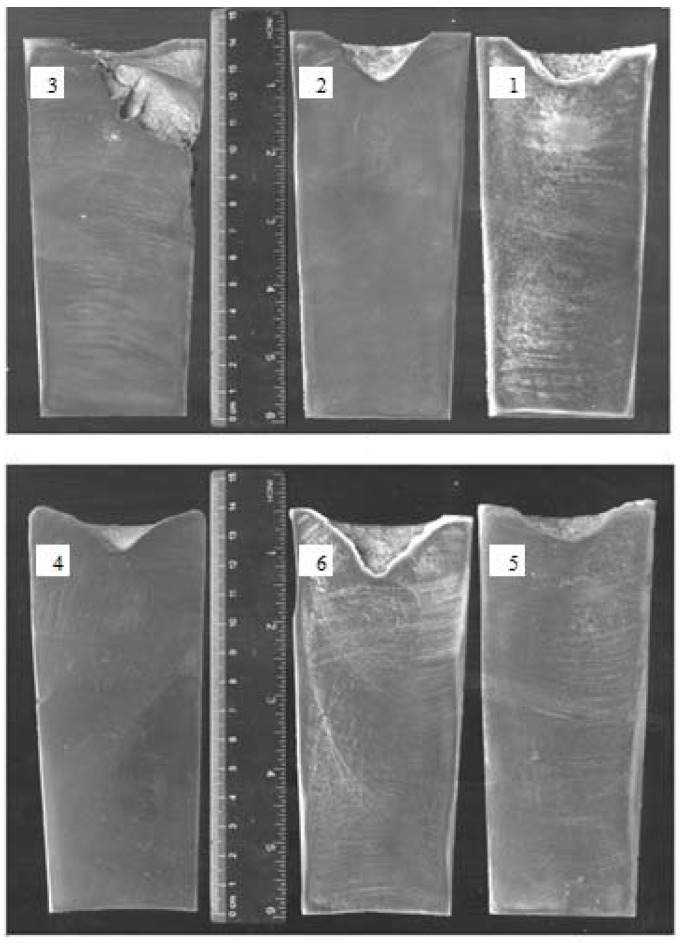
Macrostructures of 356 ingots: 1—after vibration by 100 Hz; 2—after vibration by 150 Hz; 3—after vibration by 200 Hz; 4—after modification by ultrafine powder modifier; 5—after modification by ultrafine powder modifier followed by 100 Hz vibration; 6—after modification by ultrafine powder modifier followed by 150 Hz vibration.

Figure 6 shows the macrostructures of aluminum 356 ingots obtained by different treatment approaches in cylindrical shaped cast iron molds.

In Figure 6, it is clearly seen that the shrinkage defects with a high porosity are present in the alloy subjected to 200 Hz vibration treatment. The alloy subjected to 150 Hz vibration treatment has 18 mm deep regular-shaped shrinkage with numerous 3–10 mm long cracks on the lateral surface with no detected porosity. The alloy subjected to 100 Hz vibration treatment has the same 18 mm deep regular-shaped shrinkage but with numerous short cracks of 2–8 mm long.

The macrostructures of ingots subjected to modification, modification followed by 150 Hz vibration treatment, and modification followed by 100 Hz treatment have 10, 25 and 12 mm deep regular-shaped shrinkages. Furthermore, 4–6 mm long cracks on the lateral surface and 1–3 mm long cracks in the center were detected only in alloys subjected to 150 and 100 Hz vibration treatment.

**Figure 7:**
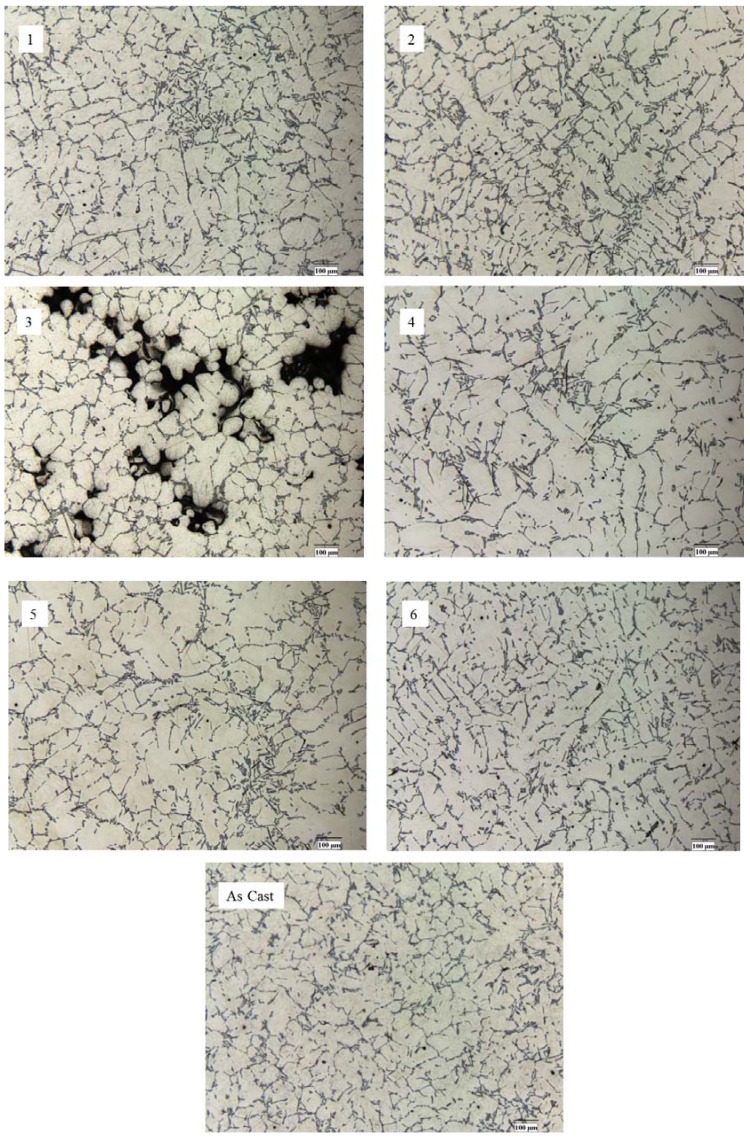
Optical microstructures of 356 alloy: 1—after vibration by 100 Hz; 2—after vibration by 150 Hz; 3—after vibration by 200 Hz; 4—after modification by ultrafine powder modifier; 5—after modification by ultrafine powder modifier followed by 100 Hz vibration; 6—after modification by ultrafine powder modifier followed by 150 Hz vibration; As cast—unmodified and untreated alloy.

Microstructures of all treated, modified and as-cast alloys are presented in Figure 7. The specimens for microstructural studies were taken from the central area of the cast and cross-sections were prepared. Image analysis was performed based on the obtained microstructures, and the calculated length of the α-Al grains and eutectic field percentage are presented in Table 4.

From the optical microscopy observation, it is evident that the microstructure became finer and typical dendrites are observed in microstructures subjected to the combined treatment (Figure 7(1,2)) compared to the unmodified alloy (Figure 7(As cast)). The high porosity microstructure observed in Figure 7(3), which was subjected to the vibration treatment by 200 Hz, is a result of applying too high of a frequency. The obtained microstructure with high differences in density of the alloy is the result of the segregation effect during metal solidification. As a result, the high porosity of the alloy is the main reason for the inability to determine its mechanical properties. In microstructures subjected to the combined approach, that is, by modification followed by vibration treatment (Figure 7(5–6)) as well as the microstructure of the alloy subjected to the modification only (Figure 7(4)), the typical α-Al grains with distributed Si network are observed. These alloys’ microstructures are identical to the as-cast alloy’s microstructure. Therefore, no significant improvements in the mechanical properties of these alloys were detected. Table 5 shows the quantification of the microstructural components based on the obtained microstructures in Figure 6.

It is evident that the average length of the α-Al grains decreased after vibration treatment by around 15%, and the eutectic phase composition increased simultaneously by 14% and even 43% after applying 100 Hz and 150 Hz vibration treatment, respectively. Vibration treatment is a type of physical influence, and is a main factor in changes of the critical points location in the Al-Si phase diagram which moves toward eutectic crystallization. While the modifier is added, the critical points come back and the alloy crystalizes in a near equilibrium state. Therefore, the eutectic phase composition increases after vibration treatment and exhibits no significant changes when the modifier is added into the melt.

**Figure 8:**
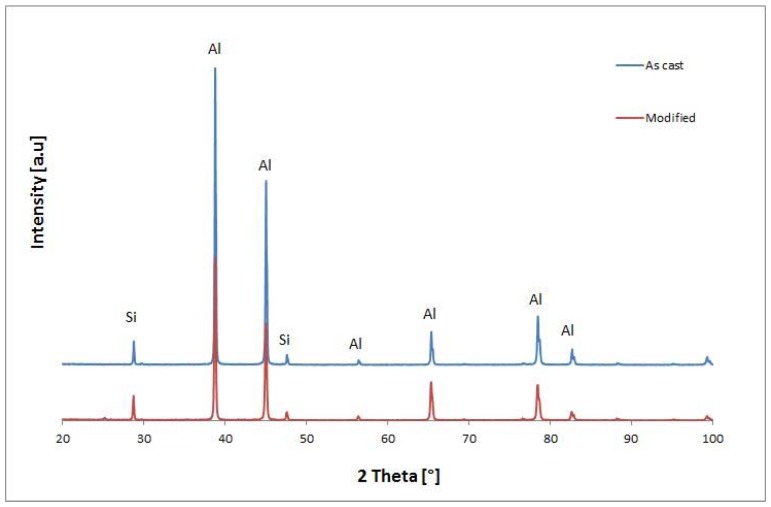
X-ray diffraction (XRD) patterns of Al 356 alloy before and after the modification process by the modifier based on the mixture of ultrafine oxide powders.

XRD analysis was performed to evaluate any structural changes in the alloy after the modification process based on the mixture of ultrafine oxide powders. The XRD patterns of the combined treatment, modified, and unmodified alloys are compared and illustrated in Figure 8. 

The XRD results indicate no difference in phase composition of a detectable amount. That’s resulting of a low amount of the added powder which affected the structure only.

From a technological point of view, this is a valuable result because the chemical composition of the initial alloy does not change after such modifications, and as such permits usage of the modifier according to customer requirements.

## 4. Conclusions

The effect of vibration, modification, and a combined treatment on aluminum casting 356 alloy structure and mechanical properties has been investigated. The following was concluded:

1. Study of the mechanical properties revealed that the alloys subjected to 100 Hz and 150 Hz vibration treatment showed the highest properties, as the tensile and the yield strengths improved by 20% and 10% respectively.
2. The densities of the alloys subjected to the vibration treatment varied in the range of 2.5–3.7% as a result of the presence of segregation areas in the obtained cast ingots.
3. The use of relatively high vibration frequencies (200 Hz) caused the formation of a high porosity microstructure and therefore caused major defects and the failure of the alloy.
4. The microstructural studies followed by an image analysis evaluated that the alloys subjected to the vibration treatment showed the refinement of α-Al grains with an increase in the Si network area around them.
5. XRD results demonstrated that there was no formation of any new phase after the modification process.

## Figures and Tables

**Table 1:** Aluminum 356 alloy chemical composition (wt %).

Si	Mg	Fe	Cu	Zn	Ni	Al
8.35	0.39	0.48	0.05	0.03	0.01	Base

**Table 2:** Homemade “Typhoon-Z” modifier chemical composition (wt %).

Al_2_O_3_	Fe_2_O_3_	CaO	K_2_O + Na_2_O	MgO	C	TiO_2_	S	FeO	SiO_2_
25	8	4	4	3	1.5	1	1	0.5	Base

**Table 3:** Experimental procedures list.

Sample No.	Vibration Frequency (Hz)	Modifier
1	100	-
2	150	-
3	200	-
4	-	+
5	100	+
6	150	+
As cast	-	-

**Table 4:** Density measurements performed on 356 Al alloy: 1—after vibration by 100 Hz; 2—after vibration by 150 Hz; 3—after modification by ultrafine powder modifier followed by 200 Hz vibration; 4—after modification by ultrafine powder modifier; 5—after modification by ultrafine powder modifier followed by 100 Hz vibration; 6—after modification by ultrafine powder modifier followed by 150 Hz vibration; As cast—unmodified and untreated alloy.

Sample No.	Average Density (g/cm^3^)
As cast	2.752 ± 0.015
1	2.733 ± 0.004
2	2.786 ± 0.047
3	2.784 ± 0.020
4	2.736 ± 0.000
5	2.768 ± 0.027
6	2.734 ± 0.002

**Table 5:** Quantification results based on microstructure image analysis: 1—after vibration by 100 Hz; 2—after vibration by 150 Hz; 4—after modification by ultrafine powder modifier; 5—after modification by ultrafine powder modifier followed by 100 Hz vibration; 6—after modification by ultrafine powder modifier followed by 150 Hz vibration; as cast—unmodified and untreated alloy.

	As Cast	1	2	4	5	6
Length of α-Al grains (μm)	38.97	33.64	32.67	38.73	35.07	36.19
Eutectic phase area (%)	15.18	17.27	21.73	12.51	16.61	13.32
